# Emergency Department Utilization by Veterans for Low-Acuity Conditions After Virtual Care Expansion

**DOI:** 10.1001/jamanetworkopen.2025.45696

**Published:** 2025-11-26

**Authors:** Anu Ramachandran, Linda Diem Tran, Steven Asch, Derek Boothroyd, Amy Justice, Louise Davies, Anita Vashi

**Affiliations:** 1 Center for Innovation to Implementation, Veterans Affairs (VA) Palo Alto Health Care System, Menlo Park, California; 2 Department of Health Policy, Stanford University, Stanford, California; 3 Health Economics Resource Center, VA Palo Alto Health Care System, Menlo Park, California; 4 Department of Surgery, Stanford School of Medicine, Stanford University, Stanford, California; 5 Division of Primary Care and Population Health, Stanford University, Stanford, California; 6 Quantitative Sciences Unit, Stanford University, Stanford, California; 7 VA Connecticut Healthcare System, West Haven; 8 Yale School of Medicine and Public Health, Yale University, New Haven, Connecticut; 9 Section of Otolaryngology, Department of Surgery, VA Affairs Medical Center, White River Junction, Vermont; 10 Department of Surgery, Geisel School of Medicine at Dartmouth, Hanover, New Hampshire; 11 Department of Emergency Medicine, University of California, San Francisco, San Francisco; 12 Department of Emergency Medicine (Affiliated), Stanford University, Stanford, California

## Abstract

**Importance:**

Low-acuity conditions account for 60% of emergency department (ED) visits within the Veterans Affairs Health System (VA). The COVID-19 pandemic led to rapid expansion of VA virtual care, but the impact on low-acuity ED utilization remains unknown.

**Objective:**

To examine patterns in low-acuity VA ED visits before and after the expansion of virtual care and to assess whether changes in alternative care use were associated with ED utilization.

**Design, Setting, and Participants:**

This retrospective cross-sectional study used an interrupted time series analysis to evaluate monthly patterns in low-acuity ED visits from March 1, 2017, through February 28, 2023, using VA Corporate Data Warehouse encounter data supplemented with administrative claims from the Office of Integrated Veteran Care. Participants included veterans presenting for acute unscheduled care at EDs within the VA. Secondary analyses included patient characteristics of low-acuity ED users and associations between ED and alternative care utilization. Data were analyzed from March 1, 2024, to August 30, 2025.

**Exposures:**

System-wide expansion of VA virtual care from March 15 to May 30, 2020

**Main Outcomes and Measures:**

The primary outcome consisted of monthly counts of low-acuity visits to VA EDs.

**Results:**

The sample included 10 364 893 VA ED visits made by 2 592 998 veterans (mean [SD] age, 60.8 [16.1] years; 9 284 407 [89.6%] by male patients). Low-acuity ED utilization dropped by 24 514 (95% CI, 12 351-36 677) visits (*P* < .001) in March 2020. From May 2020 onward, low-acuity visits increased by a mean of 7863 (95% CI, 93-15 633) per month (*P* = .047) but remained 12.4% below baseline by the end of the study period. High-acuity ED visits showed similar patterns. No association was observed between virtual care utilization and reductions in ED use for selected low-acuity conditions.

**Conclusions and Relevance:**

In this cross-sectional, national study of ED visits within the VA, low-acuity utilization declined following the expansion of virtual care; however, evidence of direct substitution was not detected. These findings highlight shifting care patterns within the VA and underscore the need for further research on quality, access, and system-level outcomes.

## Key Points

**Question:**

How did emergency department (ED) utilization for low-acuity conditions change following the expansion of virtual care within the Veterans Affairs Health System (VA)?

**Findings:**

In this cross-sectional study of 10 364 893 ED visits in the VA, low-acuity ED visits decreased rapidly in March 2020, followed by a partial rebound after the expansion of virtual care, but remained 12% below baseline by February 2023.

**Meaning:**

These findings suggests a shift toward alternative care sites for nonurgent needs within the VA; however, further research is needed to assess the extent to which virtual care can substitute for low-acuity ED care and its impact on access and outcomes.

## Teaser

This cross-sectional study evaluates patterns of emergency department (ED) utilization by US veterans for low-acuity conditions during and after the expansion of virtual care within the Veterans Affairs Health System.

## Introduction

Emergency department (ED) utilization has steadily increased, and a growing proportion of visits are for low-acuity conditions, defined as nonemergent medical problems or care related to chronic conditions.^1,2^ Nationally, an estimated 25% to 38% of ED visits are low acuity, driven by perceived urgency, limited access to alternative care sites, and clinician referrals.^1,3,4^ Excess low-acuity visits negatively affect ED care through longer wait times, overcrowding, or unnecessary testing and result in increased health care spending.^5,6,7^ Given these challenges, understanding how patients utilize EDs vs other settings for low-acuity care is critical.

The Veterans Affairs Health System (VA), the largest integrated health care system in the US, is no exception. The VA operates 111 EDs across the US, and as many as 60% of visits are estimated to be of low acuity (A.R. and J. Seidenfeld, MD, MHS; unpublished data; August 2025). The COVID-19 pandemic reduced ED utilization within the VA and accelerated the expansion of virtual care.^8,9^ As an early adopter, the VA scaled rapidly: within 3 months of the pandemic’s onset, telephone visits increased by 131% and video visits by 1080%.^10,11^ Additional initiatives included clinical contact centers offering telephone triage and symptom assessment and tele-emergency care pilots enabling remote virtual evaluation by emergency physicians.^12,13^ Collectively, these programs expanded venues for veterans to seek care for unscheduled, low-acuity conditions.

VA utilization patterns are now stabilizing following pandemic-related disruptions, large-scale virtual care expansion, and implementation of policies such as the VA MISSION Act, which increased veterans’ access to non-VA services. Understanding shifts in low-acuity ED use in this evolving context has important implications for resource allocation, patient experience, and safety. Prior studies suggest that virtual visits can offer safe and effective alternatives to ED care for low-acuity conditions.^14^ While some research indicates that increased virtual care may reduce downstream ED utilization, others hypothesize that virtual care could increase ED referrals as in-person availability is reduced.^15,16^ The degree to which the VA’s virtual care expansion has influenced low-acuity ED utilization, particularly beyond the initial onset of the COVID-19 pandemic, remains unclear.

To address this gap, we evaluated patterns of low-acuity ED utilization during and after the VA’s virtual care expansion using a newly developed diagnosis-based classification system. We compared demographic and geographic characteristics of veterans who visited the ED for low-acuity conditions before and after expansion. Finally, we analyzed utilization patterns and modeled associations for selected low-acuity conditions across 3 alternative care settings: VA in-person primary care, VA virtual care programs, and non-VA (community) acute care.

## Methods

### Study Design, Setting, and Participants

This cross-sectional study used a national sample of all VA ED visits between March 1, 2017, and February 28, 2023, among veterans 18 years or older. Secondary analysis included visits to VA primary care, VA virtual care, and non-VA community care. The Stanford University Institutional Review Board approved the study and waived informed consent because this research could not practically be carried out without the waiver and posed minimal risk. The study followed the Strengthening the Reporting of Observational Studies in Epidemiology (STROBE) reporting guideline.

### Exclusions

We excluded ED visits with diagnosis codes that could not be classified as high or low acuity (eg, left without being seen; *International Statistical Classification of Diseases, Tenth Revision* [*ICD-10*], code Z5321) and diagnoses with fewer than 10 visits per month across all VA EDs. COVID-19–related visits were excluded to account for pandemic-related utilization. We also excluded upper respiratory tract infections because of their overlap with COVID-19 diagnoses, pandemic-related declines in incidence, and seasonal fluctuations, which made pre-expansion and postexpansion comparisons less reliable.

### Exposure: Virtual Care Expansion

Virtual care expansion was defined as occurring from March to May 2020, based on prior VA evaluations.^11^ On March 15, 2020, VA leadership directed facilities to convert in-person visits to virtual visits when appropriate and implemented polices to support telephone and video encounters.^17^ By early June 2020, virtual care rose from 14% to 58% of all outpatient encounters, where it plateaued.^18^ Three pre-expansion baseline years were defined as March 1, 2017, to February 29, 2020, with subsequent postexpansion years 1 (March 1, 2020, to February 28, 2021), 2 (March 1, 2021, to February 28, 2022), and 3 (March 1, 2022, to February 28, 2023).

### Outcome: Low-Acuity ED Utilization

Low-acuity ED visits were defined as discharged encounters for which the primary diagnosis code was classified as low acuity. Acuity designation was based on a diagnosis code list developed through an iterative process that built on established ED classification methods, adapted for veteran populations. Related low-acuity diagnoses were grouped; details are provided in eTable 1 in Supplement 1.

For secondary analyses, we examined a subset of 20 low-acuity conditions: the 10 diagnoses with the largest absolute decline in visit counts and the 10 with the largest relative decline between the baseline and postexpansion periods. This subset was used to explore whether care shifted across settings for conditions most affected by the expansion. We examined utilization across (1) VA in-person primary care, (2) VA virtual care (telephone or video primary care visits, clinical contact center encounters, or tele-emergency care), and (3) community (non-VA) acute care (ED and urgent care [UC]). Encounters with matching primary *ICD-10* codes were included.

### Variables of Interest

ED visit variables included date, disposition (admission, death, or discharge), and primary *ICD-10* diagnosis. Alternative care variables included visit date, modality (in-person, telephone, or video), and primary *ICD-10* diagnosis.

Patient-level variables included age, sex, race (American Indian or Alaska Native, Asian, Black or African American, Native Hawaiian or Other Pacific Islander, and White), ethnicity (Hispanic or Latino vs non-Hispanic or non-Latino), distance to nearest VA ED, rurality, housing status, Elixhauser comorbidity score, Area Deprivation Index, VA service-connected disability status, and VA priority group (eTable 2 in Supplement 1).^19,20^ Variables were chosen based on prior work demonstrating differential access to ED or virtual care.^21,22,23^ Patient-level variables were assigned by fiscal year of the visit; if unavailable, the prior year’s values were used.

### Data Sources

All VA encounters (ED, primary care, and virtual care) and associated patient and visit variables were identified from the VA Corporate Data Warehouse, a national repository of clinical and administrative data. Stop codes were used to identify visit types and clinical groups. Community ED and UC encounters were identified from administrative claims obtained from the Office of Integrated Veteran Care. Race and ethnicity were derived from the electronic health record.

### Statistical Analysis

We calculated high- and low-acuity ED visit counts, proportion of low-acuity visits, and the most common low-acuity diagnoses. For the primary analysis, we used an interrupted time series (ITS) design to evaluate changes in monthly low-acuity ED utilization. Two intervention points were examined: March 2020 (start of the pandemic and virtual care scale-up) and May 2020 (when virtual care plateaued). The study period spanned from March 2017 to February 2023 (72 months); data were analyzed from March 1, 2024, to August 30, 2025. We adjusted for monthly seasonality and estimated Newey–West SEs to address autocorrelation and heteroskedasticity. Cumby-Huizinga tests identified autocorrelation and lag orders. ITS analyses were repeated for high-acuity visits and the 4 most common low-acuity diagnoses.

In secondary analysis, we compared the characteristics of ED users with low-acuity visits before and after virtual care expansion, using 2 years of data (baseline year 3: March 1, 2019, to February 29, 2020; postexpansion year 3: March 1, 2022, to February 28, 2023). Each patient’s first low-acuity visit in each period was included. Standardized mean differences quantified comparisons, with values of 0.1 or greater interpreted as meaningful.^24^

We also modeled associations between ED utilization within the VA and alternative community care using generalized estimating equation Poisson models for the 20 selected low-acuity diagnoses, using data from baseline year 3 and postexpansion year 3. Data were grouped by VA facility (the facility to which a patient is assigned for their routine care), and each observation represented monthly counts of visits by patients assigned to each facility at 1 of 4 sites: VA EDs, VA virtual care, VA in-person primary care, and community care. The dependent variable was monthly VA ED visits and the independent variable of interest was VA virtual care visits. An offset was included for number of patients per facility. An interaction term between each of the 3 alternative care sites and the postexpansion indicator tested whether associations differed after virtual care expansion. Effect estimates were scaled to reflect the change in VA ED visits associated with a 10% increase in alternative care utilization. As a sensitivity analysis, we estimated a generalized linear model with a random effect for VA site. ITS analyses were conducted in Stata, version 18.0 (StataCorp LLC).^25^ All other analyses were performed in R, version 3.6.1 (R Program for Statistical Computing).^26^ Two-sided *P* < .05 was considered statistically significant.

## Results

A total of 2 592 998 unique veterans made 10 364 893 ED visits during the study period, of which 5 631 202 (54.3%) were low acuity (Table 1). The mean (SD) age was 60.8 (16.1) years; 9 284 407 visits (89.6%) were by male patients and 1 080 486 (10.4%) were by female patients. A total of 85 056 visits (0.8%) were by American Indian or Alaska Native patients, 81 685 (0.8%) by Asian patients, 2 937 678 (28.3%) by Black or African American patients, 866 238 (8.4%) by Hispanic or Latino patients, 82 919 (0.8%) by Native Hawaiian or Other Pacific Islander patients, and 6 602 407 (63.7%) by White patients; 575 148 patients (5.5%) were missing race and ethnicity data.

**Table 1.** Characteristics of Study Population

Characteristic	Visit type, No. (%)		
	Low-acuity (n = 5 631 202)	High-acuity (n = 4 733 691)	Overall (N = 10 364 893)
Age, mean (SD), y	58.4 (16.2)	63.6 (15.6)	60.8 (16.1)
Age category, y			
18-45	1 315 293 (23.4)	690 649 (14.6)	2 005 942 (19.4)
46-65	2 172 761 (38.6)	1 594 537 (33.7)	3 767 298 (36.3)
≥66	2 142 204 (38.0)	2 447 816 (51.7)	4 590 020 (44.3)
Missing	944 (0.02)	689 (0.02)	1633 (0.02)
Sex			
Female	682 047 (12.1)	395 711 (8.4)	1 077 758 (10.4)
Male	4 947 650 (87.9)	4 336 757 (91.6)	9 284 407 (89.6)
Missing	1505 (0.03)	1223 (0.03)	2728 (0.03)
Race			
American Indian or Alaska Native	45 957 (0.8)	39 099 (0.8)	85 056 (0.8)
Asian	50 545 (0.9)	31 140 (0.7)	81 685 (0.8)
Black or African American	1 724 912 (30.6)	1 212 766 (25.6)	2 937 678 (28.3)
Native Hawaiian or Other Pacific Islander	47 324 (0.8)	35 595 (0.8)	82 919 (0.8)
White	3 441 697 (61.1)	3 160 710 (66.8)	6 602 407 (63.7)
Missing	320 767 (5.7)	254 381 (5.4)	575 148 (5.5)
Ethnicity			
Hispanic or Latino	495 039 (8.8)	371 199 (7.8)	866 238 (8.4)
Non-Hispanic or non-Latino	5 020 738 (89.2)	4 266 757 (90.1)	9 287 495 (89.6)
Missing	115 425 (2.0)	95 735 (2.0)	211 160 (2.0)
Unhoused	673 475 (12.0)	678 499 (14.3)	1 351 974 (13.0)
Elixhauser comorbidity score, mean (SD)^a^	4.33 (2.92)	6.0 (3.4)	5.2 (3.3)
VA service connection			
0	1 882 118 (33.4)	1 832 658 (38.7)	3 714 776 (35.8)
1-49	1 109 234 (19.7)	867 626 (18.3)	1 976 860 (19.1)
50-99	1 698 820 (30.2)	1 173 797 (24.8)	2 872 617 (27.7)
100	917 275 (16.3)	840 665 (17.8)	1 757 940 (17.0)
Missing or NA	23 755 (0.4)	18 945 (0.4)	42 700 (0.4)
VA priority category			
Highly disabled	2 831 270 (50.3)	2 342 053 (49.5)	5 173 323 (49.9)
Low to moderate disability	1 073 961 (19.1)	826 494 (17.5)	1 900 455 (18.3)
Low income	1 149 389 (20.4)	1 107 059 (23.4)	2 256 448 (21.8)
Nondisabled, copayment required	571 142 (10.1)	454 321 (9.6)	1 025 463 (9.9)
Missing	5440 (0.1)	3764 (0.1)	9204 (0.1)
Rurality			
Highly rural	83 990 (1.5)	82 166 (1.7)	166 156 (1.6)
Rural	1 152 811 (20.5)	1 037 300 (21.9)	2 190 111 (21.1)
Urban	4 383 995 (77.9)	3 605 854 (76.2)	7 989 849 (77.1)
Missing	10 406 (0.2)	8371 (0.2)	18 777 (0.2)
Driving distance from nearest VA ED, mean (SD), miles	27.6 (34.4)	28.1 (34.5)	27.8 (34.4)
Area Deprivation Index percentile^b^			
0-24	807 834 (14.3)	646 137 (13.6)	1 453 971 (14.0)
25-49	1 513 184 (26.9)	1 219 394 (25.8)	2 732 578 (26.4)
50-74	1 733 050 (30.8)	1 437 598 (30.4)	3 170 648 (30.6)
75-100	1 478 926 (26.3)	1 285 959 (27.2)	2 764 885 (26.7)
Missing	98 208 (1.7)	144 603 (3.1)	242 811 (2.3)

Abbreviations: ED, emergency department; VA, Department of Veterans Affairs.

^a^ Includes 31 conditions, with higher scores more comorbidities.

^b^ Scores range from 0 to 100, with higher scores indicating residing in an area with higher levels of deprivation.

### Patterns in Low-Acuity ED Utilization

**Figure 1.**
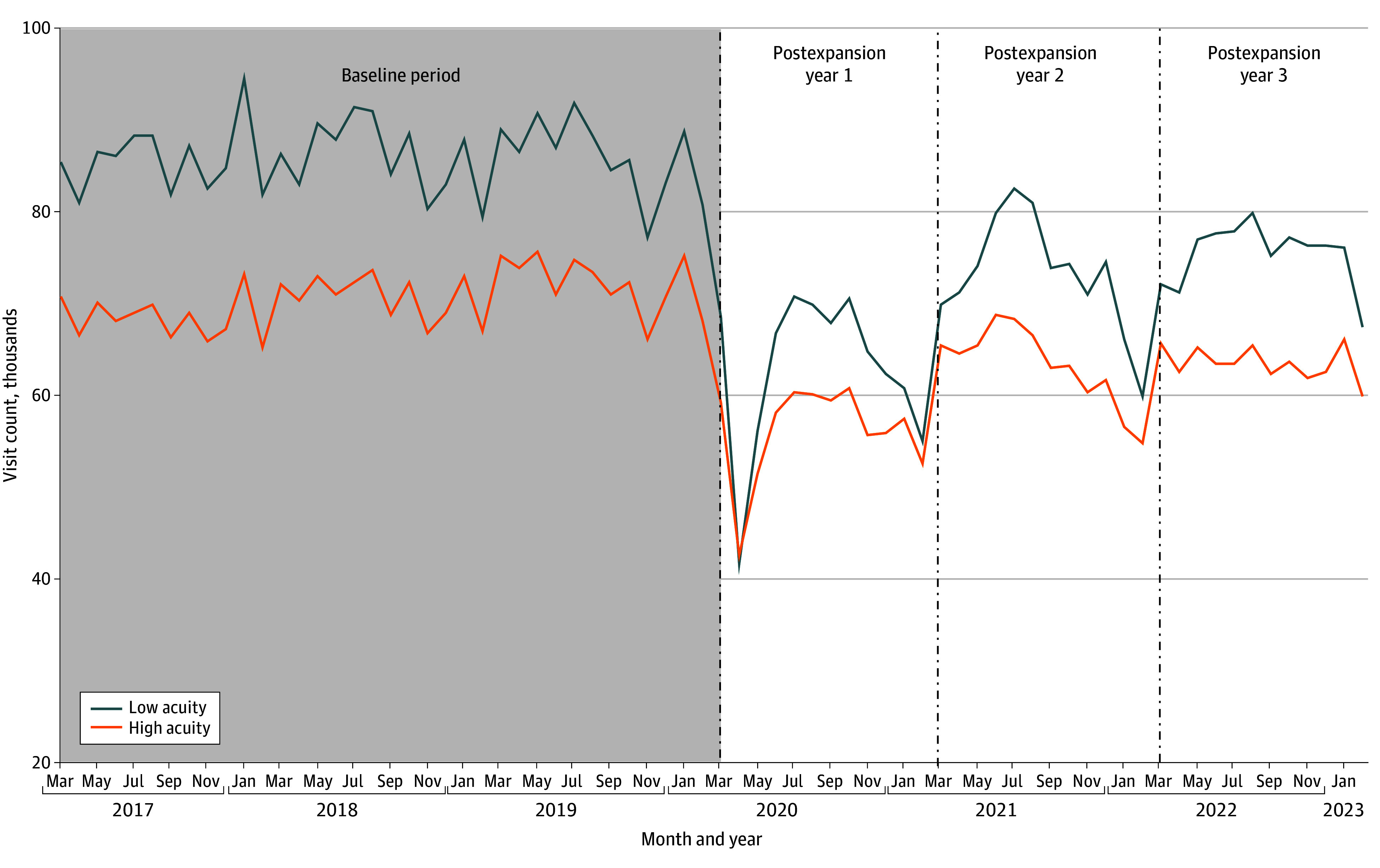
Monthly Visit Counts to Department of Veterans Affairs Emergency Departments for High- vs Low-Acuity Conditions March to May 2020 represents the expansion period.

**Figure 2.**
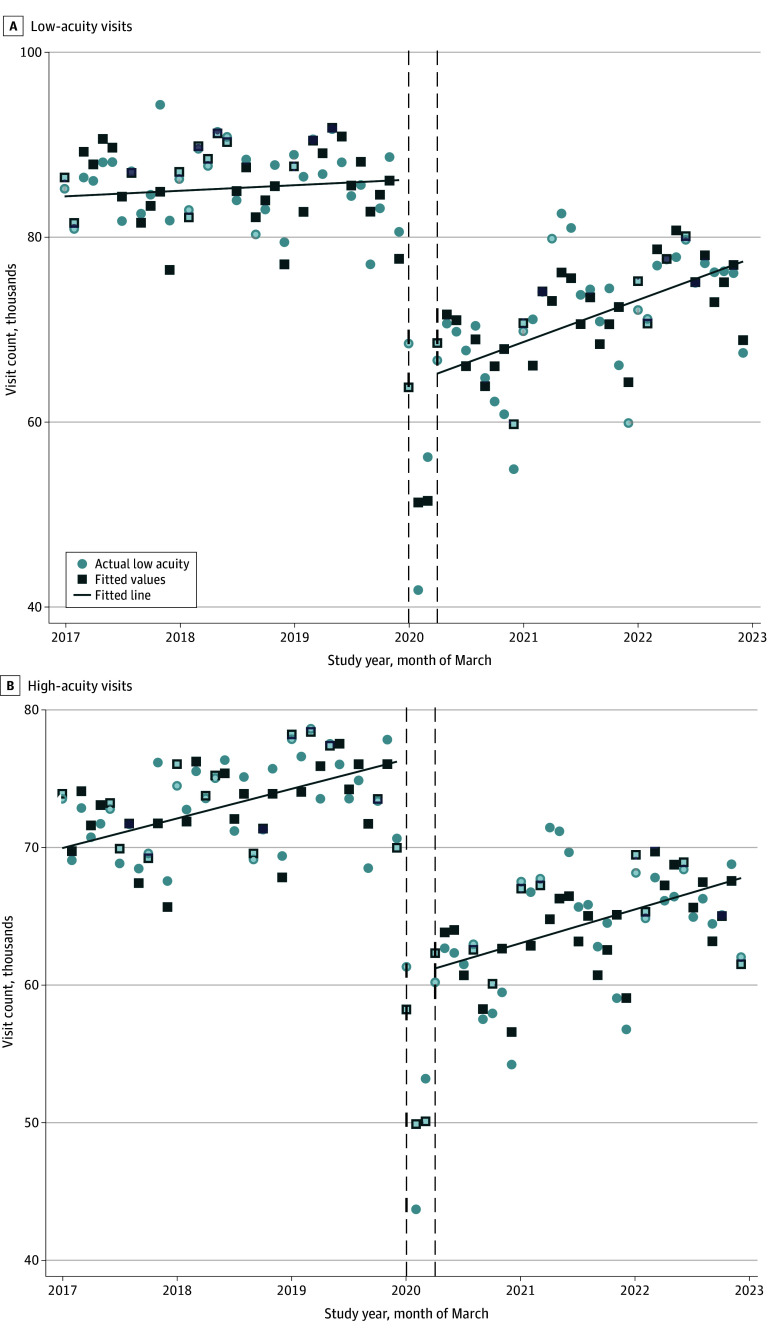
Trends in Department of Veterans Affairs Emergency Department Utilization Following Virtual Care Expansion Results were derived from interrupted time series analysis. Models include month-level adjustment for seasonality.

In the 3-year baseline period, low-acuity ED utilization was steady, with a median of 86 742 (IQR, 84 438-89 046) low-acuity visits per month by baseline year 3 (Figure 1). Starting in March 2020, following the COVID-19 pandemic declaration and the onset of VA virtual care expansion, low-acuity ED visits decreased by a median of 24 514 (95% CI, 12 351-36 677) visits in that month (*P* < .001) (Figure 2A). After May 2020, low-acuity visits increased by a median of 7863 (95% CI, 93-15 633) visits per month (*P* = .047) but remained 12.4% below baseline at the end of the study period (904 394 visits in postexpansion year 3 vs 1 032 898 in baseline year 3).

High-acuity visits followed a similar pattern, decreasing by a median of 22 197 (95% CI, 16 990-27 263) visits in March 2020 (*P* < .001) and increasing by 4180 (95% CI, 46-8314) visits per month (*P* = .05) in the postexpansion period (Figure 2B). No significant difference was found in the postexpansion slopes between high- and low-acuity visits (*P* = .09).

Utilization trends varied across the 4 low-acuity conditions examined (eFigure in Supplement 1). Median visits for low back pain decreased sharply in March 2020 (−2913 [95% CI, −2187 to −3639]; *P* < .001) and showed a flat postexpansion slope (361 [95% CI, −217 to 939] visits per month; *P* = .22). Median visits for knee pain showed a similar trend with a sharp decrease in March 2020 (−1378 [95% CI, −1072 to −1685]; *P* < .001) and a flat slope thereafter (124 [95% CI, −120 to 370] visits/mo; *P* = .31). Median visits for urinary tract infections declined initially (−892 [95% CI, −668 to −1115]; *P* < .001) but increased over time (683 [95% CI, 295-1072] visits/mo; *P* = .001), approaching baseline levels. Median visits for cellulitis also decreased initially (−874 [95% CI, −628 to −1120]; *P* < .001), with a modest positive postexpansion slope (186 [95% CI, 4-370] visits/mo; *P* = .046).

Frequent low-acuity diagnoses were stable across the study period, with low back pain the most common (8.1% of low-acuity visits), followed by knee pain (3.9%), urinary tract infection (3.5%), and cellulitis (3.2%). Among low-acuity ED visits, the largest relative reductions were observed for major depression (42.4%), gastroenteritis (38.3%), and conjunctivitis (35.6%). The largest absolute reductions were for low back pain (approximately 18 000 fewer visits per year), knee pain (approximately 11 000 fewer visits), and cellulitis (approximately 4300 fewer visits).

### Changes in Patient Characteristics

Comparing low-acuity ED users in postexpansion year 3 vs baseline year 3 (eTable 3 in Supplement 1), a higher proportion of low acuity users had 100% VA service connection (118 178 of 584 039 [20.2%] vs 96 174 of 658 461 [14.6%]) and were classified as highly disabled (321 320 of 584 039 [55.0%] vs 316 983 of 658 461 [48.1%]) after expansion. Low-acuity users were less medically complex (mean [SD] Elixhauser comorbidity scores, 3.8 [2.5] vs 4.2 [2.9]) and less likely to have low income (91 021 of 584 039 [15.6%] vs 137 379 of 658 461 [20.9%]). No significant differences were observed by age, sex, housing status, or region.

### Alternative Care Sites for Low-Acuity Conditions

**Figure 3.**
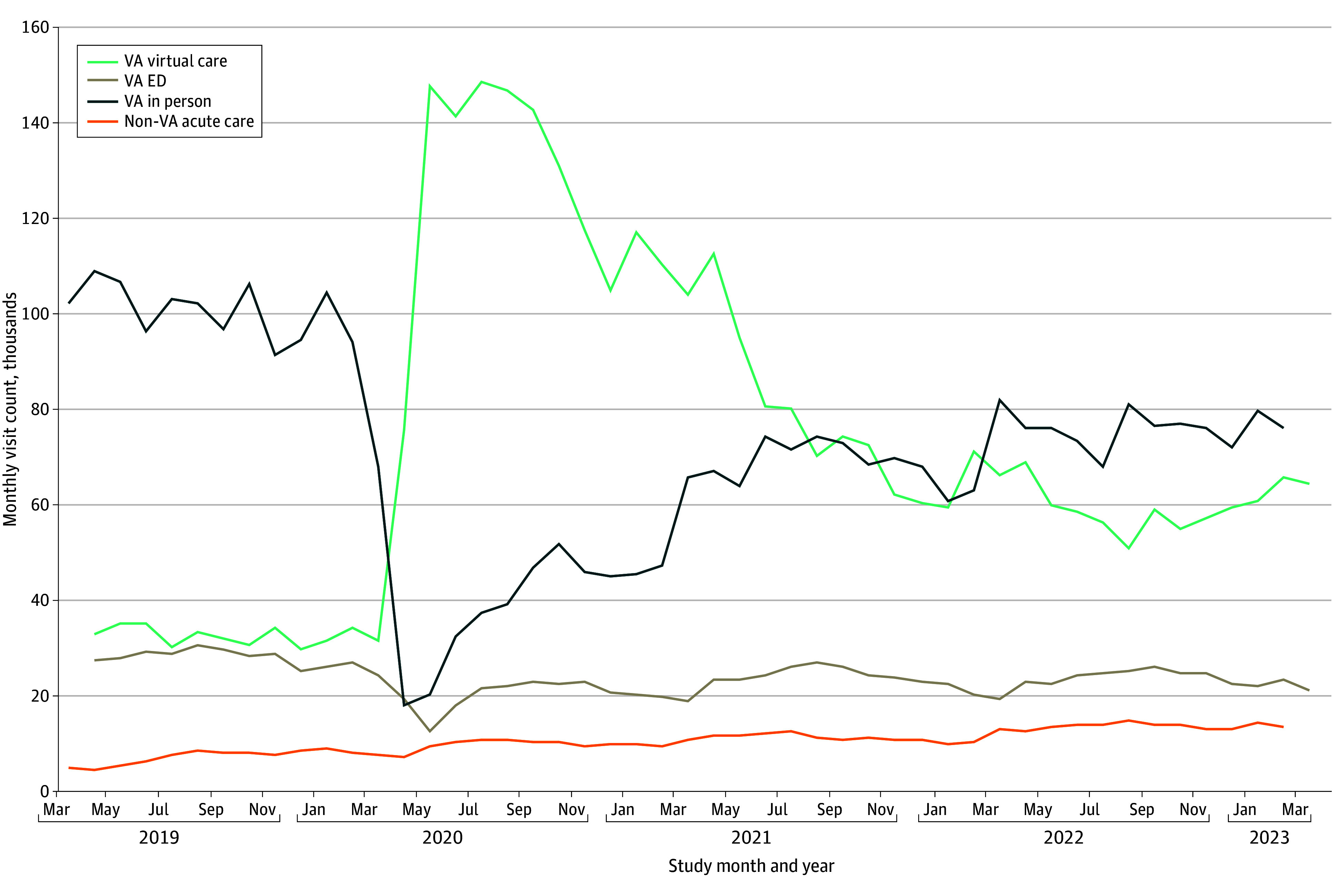
Monthly Counts of Visits for Selected Low-Acuity Diagnoses, by Care Site ED indicates emergency department; VA, Department of Veterans Affairs.

Figure 3 shows monthly visit counts to VA EDs, in-person primary care, virtual care, and community care for the 20 selected low-acuity diagnoses. Total visits across all locations increased modestly from baseline year 3 (2 016 275) to postexpansion year 3 (2 076 077). Most virtual visits occurred via telephone primary care (72.4%). Virtual care use increased sharply during the initial scale-up and remained above baseline, while in-person primary care and VA ED visits declined. Community acute care utilization increased but remained the lowest-volume site of low-acuity care.

### Association Between Alternative Care Utilization and VA ED Visits

Table 2 summarizes generalized estimating equation model results for the 20 selected diagnoses. After virtual care expansion, a 10% increase in virtual care utilization at a VA facility was not associated with a significant change in ED utilization (incidence rate ratio, 0.998 [95% CI, 0.996-1.006]; *P* = .67). Sensitivity analyses using a generalized linear model produced similar findings (eTable 4 in Supplement 1).

**Table 2.** Estimated Change in Department of Veterans Affairs Emergency Department Utilization Associated With a 10% Increase in Alternative Care^a^

Alternative care	IRR (95% CI)	*P* value^b^
Virtual visits (10% increase)	0.998 (0.996-1.006)	.67
Community visits (10% increase)	0.985 (0.980-0.991)	<.001
In-person visits (10% increase)	1.031 (1.026-1.033)	<.001

Abbreviation: IRR, incidence rate ratio.

^a^ Month-level estimates from generalized estimating equation Poisson models. Models include clustering by Department of Veterans Affairs facility, offset for number of enrollees per facility, and interactions for pre-expansion vs postexpansion periods of virtual care.

^b^ Calculated via Wald test.

## Discussion

We examined trends in low-acuity ED utilization following expansion of virtual care by the VA. Visits decreased sharply from March to May 2020, coinciding with the onset of the COVID-19 pandemic and the systemwide shift to virtual care. Utilization rebounded afterward but remained 12.4% lower than baseline levels 3 years later. High-acuity visits showed similar patterns, suggesting that overall VA ED use declined rather than shifting away from ED use for low-acuity conditions. Secondary analysis demonstrated increased utilization of virtual care and community care settings for select low-acuity conditions over the same period. However, we did not observe an association between ED use and alternative care utilization to indicate direct substitution. These findings suggest that while virtual care provides an additional access point, its role in reducing ED reliance likely depends on diagnosis, patient preference, policy environment, and whether virtual care can fully address the acute care need.

Utilization trends varied by condition. Visits for urinary tract infections and cellulitis rebounded more strongly, possibly reflecting acute symptom onset or progressing or worsening symptoms, or because they may present with more need for diagnostic testing or prescriptions. These findings suggest opportunities to divert care away from the ED when appropriate through patient education, enhanced access to same-day primary care, or refined call center triage protocols. In contrast, visits for low back pain and knee pain showed more sustained reductions in ED utilization. These conditions often involve chronic or subacute presentations, with management strategies such as self-care, physical therapy referrals, and symptom relief that may be more amenable to virtual care interventions. The decline in ED visits for musculoskeletal pain may reflect growing acceptance of virtual care as a feasible alternative, consistent with prior studies showing the effectiveness of virtual management for these conditions.^27,28^ Future research should broaden condition-specific analyses to identify which low-acuity presentations are more or less adaptable to alternative care outside of the ED.

Patients with low-acuity ED use after virtual care expansion were less medically complex and more likely to have greater connection to VA services. Veterans with more medical comorbidities may have been hesitant to seek in-person ED care following the COVID-19 pandemic. The lower share of low-income veterans among postexpansion ED users suggests possible financial barriers to ED use or greater uptake of virtual options in this group, consistent with targeted VA tablet distribution programs.^18,29^ The higher share of highly disabled veterans could indicate challenges in sustaining virtual care in this population or reflect referrals from virtual visits to the ED for further evaluation. These shifts underscore the importance of evaluating how patient subgroups navigate evolving access points and whether virtual care differentially influences ED use across vulnerable populations.

Virtual care utilization increased for selected low-acuity diagnoses, persisting through February 2023. Despite this growth, we found no significant association between virtual care use and VA ED visits across the 20 conditions analyzed, suggesting that virtual care expanded access rather than replaced ED encounters at a population level. Rather than diverting patients from the ED, availability of virtual care may have led some veterans to use it for conditions they would not otherwise have sought care for. For others, virtual care encounters may have generated new referrals to EDs by identifying cases requiring further evaluation.

Community care use also increased slightly over the study period for selected low-acuity conditions, a trend likely influenced by concurrent policy changes. The 2019 MISSION Act expanded access for veterans to community EDs, and a new urgent care benefit introduced the same year created another option for low-acuity conditions.^30^ Early evaluations showed limited uptake of the VA urgent care benefit, but subsequent studies have documented increasing utilization of community acute care over time.^9,31,32,33^ While most community ED visits are for high-acuity conditions, community UC facilities offer an important alternative to VA EDs for low-acuity care, particularly for veterans who live farther away from VA facilities. These policy changes, alongside VA’s virtual care expansion, likely contributed jointly to observed reductions in ED utilization.

Understanding how veterans choose among VA EDs, virtual care, and community care for low-acuity conditions should be a priority for future work. Prior evaluations of tele-emergency care in the VA have demonstrated reductions in ED utilization, hospitalizations, and costs among patients using the service.^15,33^ However, in our study, tele-emergency visits comprised a small fraction of overall virtual encounters, likely due to the newness of the program and limited veteran awareness.^34^ As tele-emergency care continues to expand, ongoing assessment of its impact on national VA ED utilization will be important to guide policy and resource allocation. Future research should also explore veteran decision-making under evolving policy environments to better inform patient-centered acute care delivery.

### Strengths and Limitations

Our analysis has several strengths, including use of a novel code set for identifying low-acuity ED visits, leveraging a large national dataset, and a 6-year study window spanning the MISSION Act, COVID-19 pandemic, and virtual care expansion. However, there are important limitations to consider. First, classifying visits as low acuity based on *ICD-10* codes and discharge disposition does not imply inappropriate ED use. Factors such as symptom severity, medical comorbidities, and access to care influence care-seeking decisions and are not captured by diagnosis-based classifications.^35^ Reducing low-acuity ED visits should not be considered inherently positive, as the ED remains a critical access point for unscheduled, acute care needs, particularly among disadvantaged populations.^36^ Second, we did not assess all potential alternatives, such as VA UC centers, although their limited availability (31 nationwide) reduces their system-level impact. Finally, while virtual care use rose as ED visits declined, our models do not provide evidence of direct substitution.

## Conclusions

In this national, cross-sectional study, low-acuity ED utilization declined after the VA’s expansion of virtual care. While shifting low-acuity care away from ED settings toward virtual options may improve the value and efficiency of services, questions remain about the effects on quality and patient satisfaction. Further research should be directed at exploring patient- and system-level factors that influence care-seeking decisions for low-acuity conditions. These insights will be critical in optimizing veteran-centered acute care delivery in an evolving health care landscape.

## Notes

Supplement 1. **eTable 1.** Diagnosis Groupings **eTable 2.** VA Definitions for Demographic Variables: Race, Ethnicity, Service Connection, Priority Group, ADI, and Elixhauser Comorbidity Scores **eTable 3.** Characteristics of Low-Acuity ED Users Before and After Virtual Care Expansion **eTable 4.** Sensitivity Analysis of Estimated Change in VA ED Utilization Following a 10% Increase in Alternative Care Use **eFigure.** Trends in ED Utilization Following Virtual Care Expansion Among 4 Low-Acuity Diagnoses

Supplement 2.
Data Sharing Statement

## References

[1] Uscher-Pines L, Pines J, Kellermann A, Gillen E, Mehrotra A. Emergency department visits for nonurgent conditions: systematic literature review. Am J Manag Care. 2013;19(1):47-59. 23379744 PMC4156292

[2] Sun R, Karaca Z, Wong HS. Trends in hospital emergency department visits by age and payer, 2006-2015. In: *Healthcare Cost and Utilization Project (HCUP) Statistical Briefs*. Agency for Healthcare Research and Quality; 2006. Accessed December 14, 2022. https://www.ncbi.nlm.nih.gov/books/NBK513766/30063311

[3] Gill JM, Riley AW. Nonurgent use of hospital emergency departments: urgency from the patient’s perspective. J Fam Pract. 1996;42(5):491-496. 8642367

[4] Yoon J, Cordasco KM, Chow A, Rubenstein LV. The Relationship between same-day access and continuity in primary care and emergency department visits. PLoS One. 2015;10(9):e0135274. doi:10.1371/journal.pone.0135274 26332981 PMC4557991

[5] Schull MJ, Kiss A, Szalai JP. The effect of low-complexity patients on emergency department waiting times. Ann Emerg Med. 2007;49(3):257-264, 264.e1. doi:10.1016/j.annemergmed.2006.06.027 17049408

[6] Espinosa G, Miró O, Sánchez M, Coll-Vinent B, Millá J. Effects of external and internal factors on emergency department overcrowding. Ann Emerg Med. 2002;39(6):693-695. doi:10.1067/mem.2002.124447 12023721

[7] Baughman DJ, Waheed A, Khan MN, Nicholson JM. Enhancing value-based care with a walk-in clinic: a primary care provider intervention to decrease low acuity emergency department overutilization. Cureus. 2021;13(2):e13284. doi:10.7759/cureus.13284 33728217 PMC7955766

[8] Giannouchos TV, Biskupiak J, Moss MJ, Brixner D, Andreyeva E, Ukert B. Trends in outpatient emergency department visits during the COVID-19 pandemic at a large, urban, academic hospital system. Am J Emerg Med. 2021;40:20-26. doi:10.1016/j.ajem.2020.12.009 33338676 PMC7725055

[9] Rose L, Tran LD, Asch SM, Vashi A. Assessment of changes in US Veterans Health Administration care delivery methods during the COVID-19 pandemic. JAMA Netw Open. 2021;4(10):e2129139. doi:10.1001/jamanetworkopen.2021.29139 34648015 PMC8517744

[10] Reddy A, Gunnink E, Deeds SA, . A rapid mobilization of “virtual” primary care services in response to COVID-19 at Veterans Health Administration. Healthc (Amst). 2020;8(4):100464. doi:10.1016/j.hjdsi.2020.100464 32992109 PMC7434426

[11] Heyworth L, Kirsh S, Zulman D, Ferguson JM, Kizer KW. Expanding access through virtual care: the VA’s early experience with COVID-19. NEJM Catal. Published online July 1, 2020. doi:10.1056/cat.20.0327

[12] Kirsh S, Rovinski-Wagner C, Brass S, Williams KM, Bouchard M, Kizer KW. VA Health Connect: a clinical contact center designed to enhance access and quality of care for veterans. NEJM Catal. Published online April 19, 2023. doi:10.1056/CAT.22.0292

[13] Patel N, Spiropoulos K, Celedon M, . Tele-emergency care may improve access to emergency care resources while reducing need for in-person emergency department evaluation. Ann Emerg Med. 2022;80(4):S34. doi:10.1016/j.annemergmed.2022.08.090

[14] Hsu H, Greenwald PW, Clark S, . Telemedicine evaluations for low-acuity patients presenting to the emergency department: implications for safety and patient satisfaction. Telemed J E Health. 2020;26(8):1010-1015. doi:10.1089/tmj.2019.0193 31930952

[15] Li KY, Kim PS, Thariath J, Wong ES, Barkham J, Kocher KE. Standard nurse phone triage versus tele-emergency care pilot on veteran use of in-person acute care: an instrumental variable analysis. Acad Emerg Med. 2023;30(4):310-320. doi:10.1111/acem.14681 36757685 PMC10162445

[16] Ashwood JS, Mehrotra A, Cowling D, Uscher-Pines L. Direct-to-consumer telehealth may increase access to care but does not decrease spending. Health Aff (Millwood). 2017;36(3):485-491. doi:10.1377/hlthaff.2016.1130 28264950

[17] Connolly SL, Stolzmann KL, Heyworth L, Weaver KR, Bauer MS, Miller CJ. Rapid increase in telemental health within the Department of Veterans Affairs during the COVID-19 pandemic. Telemed J E Health. 2021;27(4):454-458. doi:10.1089/tmj.2020.0233 32926664

[18] Ferguson JM, Jacobs J, Yefimova M, Greene L, Heyworth L, Zulman DM. Virtual care expansion in the Veterans Health Administration during the COVID-19 pandemic: clinical services and patient characteristics associated with utilization. J Am Med Inform Assoc. 2021;28(3):453-462. doi:10.1093/jamia/ocaa284 33125032 PMC7665538

[19] Elixhauser A, Steiner C, Harris DR, Coffey RM. Comorbidity measures for use with administrative data. Med Care. 1998;36(1):8-27. doi:10.1097/00005650-199801000-00004 9431328

[20] Carlson LC, Kim J, Samuels-Kalow ME, . Comparing neighborhood-based indices of socioeconomic risk factors and potentially preventable emergency department utilization. Am J Emerg Med. 2021;44:213-219. doi:10.1016/j.ajem.2020.03.035 32291162

[21] Vanstone NA, Belanger P, Moore K, Caudle JM. Socioeconomic composition of low-acuity emergency department users in Ontario. Can Fam Physician. 2014;60(4):355-362. 24733328 PMC4046549

[22] Altmayer CA, Ardal S, Woodward GL, Schull MJ. Variation in emergency department visits for conditions that may be treated in alternative primary care settings. CJEM. 2005;7(4):252-256. doi:10.1017/S1481803500014391 17355682

[23] Morris DM, Gordon JA. The role of the emergency department in the care of homeless and disadvantaged populations. Emerg Med Clin North Am. 2006;24(4):839-848. doi:10.1016/j.emc.2006.06.011 16982342

[24] Austin PC. Balance diagnostics for comparing the distribution of baseline covariates between treatment groups in propensity-score matched samples. Stat Med. 2009;28(25):3083-3107. doi:10.1002/sim.3697 19757444 PMC3472075

[25] *Stata Statistical Software*. Version 18.0. StataCorp LLC; 2023. Accessed August 1, 2023. https://www.stata.com

[26] *R: A Language and Environment for Statistical Computing*. Version 3.6.1. R Core Team; 2024. Accessed August 1, 2025. https://www.R-project.org/

[27] Lovo S, Imeah B, Sari N, . Effectiveness of an interprofessional assessment and management approach for people with chronic low back disorders delivered via virtual care: a randomized controlled trial pilot intervention. Digit Health. 2024;10:20552076241260569. doi:10.1177/20552076241260569 38846367 PMC11155314

[28] Kiran T, Green ME, Strauss R, . Virtual care and emergency department use during the COVID-19 pandemic among patients of family physicians in Ontario, Canada. JAMA Netw Open. 2023;6(4):e239602. doi:10.1001/jamanetworkopen.2023.9602 37115549 PMC10148195

[29] Slightam C, Gregory AJ, Hu J, . Patient perceptions of video visits using Veterans Affairs Telehealth Tablets: survey study. J Med Internet Res. 2020;22(4):e15682. doi:10.2196/15682 32293573 PMC7191342

[30] VA MISSION Act of 2018, S.2372, 115th Cong (2017-2018). Accessed September 12, 2024. https://www.congress.gov/bill/115th-congress/senate-bill/2372/text

[31] Vashi AA, Urech T, Wu S, Tran LD. Community emergency care use by veterans in an era of expanding choice. JAMA Netw Open. 2024;7(3):e241626. doi:10.1001/jamanetworkopen.2024.1626 38457180 PMC10924239

[32] Nevedal AL, Wong EP, Urech TH, Peppiatt JL, Sorie MR, Vashi AA. Veterans’ experiences with accessing community emergency care. Mil Med. 2023;188(1-2):e58-e64. doi:10.1093/milmed/usab196 34028535 PMC8611117

[33] Vashi AA, Urech T, Wu S, . Community urgent care use following implementation of the Veterans Affairs Maintaining Internal Systems and Strengthening Integrated Outside Networks Act. Med Care. 2021;59(suppl 3):S314-S321. doi:10.1097/MLR.0000000000001549 33976082 PMC8132890

[34] Tran LD, Rose L, Suzuki K, Urech T, Vashi A. Medical advice lines offering on-demand access to providers reduced emergency department visits. Health Aff Sch. 2023;1(6):qxad079. doi:10.1093/haschl/qxad079 38756361 PMC10986286

[35] Wray CM, Junge M, Keyhani S, Smith JE. Assessment of a multi-center tele-urgent care program to decrease emergency department referral rates in the Veterans Health Administration. J Telemed Telecare. 2023;29(10):749-754. doi:10.1177/1357633X211024843 34152876

[36] Raven MC, Lowe RA, Maselli J, Hsia RY. Comparison of presenting complaint vs discharge diagnosis for identifying “nonemergency” emergency department visits. JAMA. 2013;309(11):1145-1153. doi:10.1001/jama.2013.1948 23512061 PMC3711676

