# Supplementary material for: Emergency Department Utilization by Veterans for Low-Acuity Conditions After Virtual Care Expansion
Source: JAMA Netw Open. 2025 Nov 26;8(11):e2545696. doi: 10.1001/jamanetworkopen.2025.45696 (PMC12658669; doi:10.1001/jamanetworkopen.2025.45696)
Supplement: Supplement 1. — eTable 1. Diagnosis Groupings eTable 2. VA Definitions for Demographic Variables: Race, Ethnicity, Service Connection, Priority Group, ADI, and Elixhauser Comorbidity Scores eTable 3. Characteristics of Low-Acuity ED Users Before and After Virtual Care Expansion eTable 4. Sensitivity Analysis of Estimated Change in VA ED Utilization Following a 10% Increase in Alternative Care Use eFigure. Trends in ED Utilization Following Virtual Care Expansion Among 4 Low-Acuity Diagnoses [file jamanetwopen-e2545696-s001.pdf]

Supplementary Online Content

Ramachandran A, Tran LD, Asch S, et al. Emergency department utilization by veterans for low-acuity conditions after virtual care expansion. *JAMA Netw Open.* 2025;8(11):e2545696. doi:10.1001/jamanetworkopen.2025.45696

eTable 1. Diagnosis Groupings
eTable 2. VA Definitions for Demographic Variables: Race, Ethnicity, Service Connection, Priority Group, ADI, and Elixhauser Comorbidity Scores
eTable 3. Characteristics of Low-Acuity ED Users Before and After Virtual Care Expansion
eTable 4. Sensitivity Analysis of Estimated Change in VA ED Utilization Following a 10% Increase in Alternative Care Use
eFigure. Trends in ED Utilization Following Virtual Care Expansion Among 4 Low-Acuity Diagnoses

This supplementary material has been provided by the authors to give readers additional information about their work.

© 2025 Ramachandran A et al. *JAMA Network Open*.

eTable 1. Diagnosis Groupings

Grouped Diagnosis Name	Diagnoses included
Low back pain	Low back pain<br>Low back pain, other<br>Other low back pain<br>Muscle spasm of back<br>Strain of muscle, fascia and tendon of lower back, initial encounter
Knee pain	Pain in unspecified knee<br>Pain in right knee<br>Pain in left knee
Foot pain	Pain in foot and toes<br>Pain in right foot<br>Pain in left foot<br>Pain in unspecified foot<br>Pain in left/right toe(s)
Ankle sprain	Sprain of unspecified ligament of left ankle, initial encounter<br>Sprain of unspecified ligament of right ankle, initial encounter
Shoulder pain	Pain in unspecified shoulder<br>Pain in left shoulder<br>Pain in right shoulder
Encounter for examination	Encounter for examination and observation for unspecified reason<br>Encounter for other general examination<br>Encounter for screening, unspecified<br>Encounter for general adult medical examination without abnormal findings<br>Encounter for other administrative examinations<br>Encounter for administrative examinations, unspecified
Major depression	Other recurrent depressive disorders<br>Major depressive disorder, recurrent, unspecified
Gastroenteritis	Infectious gastroenteritis and colitis, unspecified<br>Noninfective gastroenteritis and colitis, unspecified<br>Other specified noninfective gastroenteritis and colitis
Migraine	Other migraine, not intractable, without status migrainosus<br>Migraine without aura, not intractable, without status migrainosus<br>Migraine, unspecified, not intractable, without status migrainosus
Limb/joint pain	Pain in unspecified limb<br>Pain in unspecified joint
Conjunctivitis	Other conjunctivitis<br>Unspecified conjunctivitis

© 2025 Ramachandran A et al. *JAMA Network Open*.

Gout	Gout, unspecified<br>Idiopathic gout, unspecified site
Cellulitis	Cellulitis, unspecified<br>Cellulitis of left/right lower limb<br>Cellulitis of unspecified part of limb<br>Cellulitis of left/right finger<br>Cellulitis of left/right upper limb<br>Cellulitis of left/right toe<br>Cellulitis of other sites
Anxiety	Anxiety disorder, unspecified<br>Generalized anxiety disorder
Counseling	Other specified counseling<br>Counseling, unspecified
Wrist pain	Pain in right wrist<br>Pain in left wrist
Hemorrhoids	Other hemorrhoids<br>Unspecified hemorrhoids
Sciatica	Sciatica, left/right side<br>Lumbago with sciatica, left/right side<br>Sciatica, unspecified side
Alcohol abuse	Alcohol abuse, uncomplicated<br>Alcohol dependence, uncomplicated
Psychosocial issues	Problem related to unspecified psychosocial circumstances<br>Other specified problems related to psychosocial circumstances

Method: First, we identified the 200 most frequent low-acuity diagnoses over the study time period. Next, two emergency medicine physicians (AR and AAV) grouped highly-related diagnoses, particularly to consolidate lateralized musculoskeletal complaints (e.g., left vs. right) into a single diagnosis.

© 2025 Ramachandran A et al. *JAMA Network Open*.

eTable 2. VA Definitions for Demographic Variables: Race, Ethnicity, Service Connection, Priority Group, ADI, and Elixhauser Comorbidity Scores

Variable	Data Source(s)	Description / Approach	Categories
Age	VA CDW OMOP Person table	Patient's age at the time of the ED or UC visit using date of birth information from VA OMOP.<br><br>The OMOP date of birth information sources include the VA CDW's Patient table data, the Vital Status file, and manual chart review information.<br><br>Veterans < 18 years are not eligible for analysis; patients aged > 110 years are set to missing.<br><br>Additional information about the VA OMOP Common Data Model can be found at: https://www.hsrd.research.va.gov/for_researchers/cyber_seminars/archives/video_archive.cfm?SessionID=3632	• 18-45<br>• 45-64<br>• >/=65<br>• Missing
Sex	VA CDW OMOP Person table	Patient's sex from VA OMOP. The algorithm prioritizes manually reviewed sex information, and the most frequent value is used. If data method of entry is unknown, the most frequent value from the Vital Status file is used. If the value is still unknown, the most frequent value from the Patient table is used. If there is still no value, then it indicated as null.	• Male<br>• Female<br>• Missing
Race and ethnicity	VA CDW OMOP Person table	Patient's race and ethnicity from VA OMOP. We reported combined race/ethnicity categories using the VA OMOP data.<br><br>In VA OMOP, patient's race, can be either self-report or non-self-report (i.e., data acquired from VHA enrollment coordinator or clerk); OMOP contains one standard value for race and ethnicity for each PersonID.<br><br>The VA OMOP Common Data Model follows the "OMB Standards for Data on Race and Ethnicity" and the VA Data Quality Program's "CDW Ethnicity Data Report" to report ethnicity.	• Non-Hispanic White<br>• Non-Hispanic Black<br>• Non-Hispanic Other (includes Asian, AI or AN, or NH or OPI)<br>• Hispanic<br>• Missing<br><br>• White<br>• Black or AA<br>• Asian<br>• AI or AN<br>• NH or OPI<br>• Unknown<br><br>• Hispanic or Latino

© 2025 Ramachandran A et al. *JAMA Network Open*.

			• Not Hispanic or Latino<br>• Unknown
VA service-connected (SC) disability rating	VA ADUSH Enrollment File	Patient's SC disability rating recorded in the enrollment file for the FY associated with the ED or UC visit.<br><br>The rating indicates an injury or illness deemed to have been incurred or aggravated while serving in the armed forces. The Veteran's SC disability is assessed and rated for severity from 0 to 100 percent. The rating can change over time. A 0% SC disability rating is not the same thing as no SC disability rating. The rating impacts the Veteran's VA priority group assignment.	• No SC disability rating<br>• 0-49%<br>• 50-99%<br>• 100%<br>• Missing
VA priority group assignment	VA ADUSH Enrollment File	Patient's priority group assignment recorded in the enrollment file for the FY associated with the ED or UC visit.<br><br>Priority group assignment (groups 1-8) is based on military service history, disability rating, income level, Medicaid eligibility, and other benefits such as VA pension benefits. VA assigns Veterans with service-connected disabilities the highest priority. VA assigns the lowest priority to Veterans who earn a higher income and who do not have any service-connected disabilities qualifying them for disability compensation. A Veteran's priority group assignment can change if the Veteran's income changes; or the Veteran's service-connected disability gets worse, and the VA indicates a higher disability rating.<br><br>We aggregated the eight priority group assignments into four categories.<br><br>Additional information about VA priority groups can be found at: https://www.va.gov/health-care/eligibility/priority-groups/.	• Groups 1 and 4: high disability<br>• Groups 2, 3, and 6: low/moderate disability<br>• Group 5: low income<br>• Groups 7 and 8: non-disabled, copayment required
Elixhauser comorbidity score	VA CDW	A measure of the patient's illness burden using ICD-10-CM diagnosis codes recorded in VA care settings (inpatient and outpatient) and in non-VA care settings in which VA was the payer (e.g., diagnosis codes from the VA purchased care files).<br><br>We used the R package for computing comorbidity scores. It uses coding algorithms published by Quan H, et al (2005). We examined the current FY and the previous FY associated with the ED or UC visit. We reported the number of Elixhauser comorbidities for the FY that had the higher count.	31 Elixhauser conditions:<br>1. AIDS/HIV<br>2. Alcohol abuse<br>3. Blood loss anemia<br>4. Cardiac arrhythmias<br>5. Chronic pulmonary disease<br>6. Coagulopathy

© 2025 Ramachandran A et al. *JAMA Network Open*.

		Gasparini A. Comorbidity: an R package for computing comorbidity scores. J. Open Source Softw. 2018;3(23):648.<br><br>*Quan H, Sundararajan V, Halfon P, et al. Coding algorithms for defining comorbidities in ICD-9-CM and ICD-10 administrative data. Med Care. 2005;43(11):1130-9.*	7. Congestive heart failure<br>8. Deficiency anemia<br>9. Depression<br>10. Diabetes, complicated<br>11. Diabetes, uncomplicated<br>12. Drug abuse<br>13. Fluid and electrolyte disorders<br>14. Hypertension, complicated<br>15. Hypertension, uncomplicated<br>16. Hypothyroidism<br>17. Liver disease<br>18. Lymphoma<br>19. Metastatic cancer<br>20. Obesity<br>21. Other neurological disorders<br>22. Paralysis<br>23. Peptic ulcer disease, excluding bleeding<br>24. Peripheral vascular disorders<br>25. Psychoses<br>26. Pulmonary circulation disorders<br>27. Renal failure<br>28. Rheumatoid arthritis/collagen vascular diseases<br>29. Solid tumor without metastasis<br>30. Valvular disease<br>31. Weight loss
Housing status	VA CDW	The patient's housing status during the FY associated with the ED or UC visit. If the Veteran had at least one indication of being unhoused during the FY, the Veteran was indicated as being unhoused.	• Housed<br>• Unhoused

© 2025 Ramachandran A et al. *JAMA Network Open*.

		Data to determine housing status included: (1) VA outpatient visits related to housing services, health care for the unhoused, employment services, and outreach programs; (2) VA inpatient admissions to treating specialties focused on caring for unhoused Veterans (e.g., Domiciliary care); (3) a ICD-10-CM diagnosis code indicated housing instability or homelessness recorded in the VA outpatient or inpatient setting; or (4) clinical reminder documentation in the EHR indicating the Veteran is unhoused or has a housing concern.<br><br>The approach approximates the methodological approach described in the Tsai J, et al (2022).<br><br>*Tsai J, Szymkowiak D, Jutkowitz E. Developing an operational definition of housing instability and homelessness in Veterans Health Administration's medical records. PLoS One. 2022;17(12):e0279973.*	
Rurality of residential location	VA PSSG Enrollee File	The patient's rurality designation at the end of the FY for the FY associated with the ED or UC visit.<br><br>Patients living in census tracts with rural-urban commuting area codes (RUCA) scores of 1.0 or 1.1 are categorized as urban. Patients residing in census tracts of 10.0 are categorized as highly rural. Patients living in census tracts not categorized as urban or highly rural are categorized as rural.<br><br>We grouped rural and highly rural patients into one category.	• Urban<br>• Rural and highly rural<br>• Missing
US Census Region	CDW<br>VA PSSG Enrollee File	Using the patient's most recent address (state) in the FY associated with the ED or UC visit, we categorized the patient's residence into one of the four US Census Regions.	• Northeast<br>• South<br>• Midwest<br>• West
Drive time to closest VA primary care	VA PSSG Enrollee File	Driving distance (in minutes) for the VA site closest to the patient's residence address as of the end of the designated fiscal year for VA primary care services. We used the value associated with the FY of the ED or UC visit.	• 0-30<br>• >30-60<br>• >60<br>• Missing
Area Deprivation Index (ADI)	CDW<br>VA PSSG Enrollee File	We included this variable as a marker of socioeconomic deprivation. The ADI is composed of 17 measures related to education, employment, housing quality, and poverty. The ADIs are provided in national percentile rankings at the Census block group level from 1 to 100. A block	• 0-25th percentile (least deprived)<br>• 25th-50th percentile<br>• 50th-75th percentile

© 2025 Ramachandran A et al. *JAMA Network Open*.

		group with a ranking of 1 indicates the lowest level of disadvantage within the nation and an ADI with a ranking of 100 indicates the highest level of disadvantage.<br><br>In the CDW, we used the Patient level ADI table associated with the FY of the ED or UC visit.<br><br>ADI developed by Neighborhood Atlas, University of Wisconsin.<br><br>Based on previous literature (Edmonds AT, et al 2022), we dichotomized the ADI percentile rankings.<br><br>*Edmonds AT, Rhew IC, Jones-Smith J, et al. Neighborhood disadvantage, patterns of unhealthy alcohol use, and differential associations by gender, race/ethnicity, and rurality: a study of Veterans Health Administration patients. J Stud Alcohol Drugs. 2022;83(6):867-878.*	• 75th-99th percentile (most deprived)<br>• Missing
VA ED visit identification	Stop Codes	The Veterans Health Administration (VHA) uses stop codes to identify workload for all outpatient encounters, inpatient appointments in outpatient clinics and inpatient professional services. A primary stop code and a secondary stop code compose the six digit stop code pair. The first three digits represent the primary stop code. The primary stop code designates the main clinical group responsible for the care. The last three digits of the stop code contain the secondary stop code, or credit stop, which serves as a modifier to further define the primary work group. Following VHA Directive 1101.14(1) Emergency Medicine: "All patients receiving face-to-face evaluation in an ED, regardless of the severity of their illness or triage level, must be coded under a 130 stop code encounter," we used stop code 130 (in the primary stop code position) to identify VA ED visits. To ensure appropriate use of stop code 130 in the electronic health record, we also required that the visit (indicated by the stop code 130) was recorded at a VA facility with an emergency department. Our study team maintains an annual list of active VA ED facilities that is developed using VA Corporate Data Warehouse data and is validated with feedback from the VA National Emergency Medicine Office (NEMO) and VA emergency medicine staff in the field. We track when a VA ED closes temporarily (e.g., renovation) or permanently (e.g., transitions to an urgent care center) and use this information to identify eligible 130 encounters. For example, if a visit is documented as 130 at a facility that is no longer operating a VA ED, this visit is not included in our analysis.	

Abbreviations: ADUSH, Assistant Deputy Under Secretary for Health; AHA, American Hospital Association; AN, Alaskan Native; AI, American Indian; CDW, Corporate Data Warehouse; ED, emergency department; FY, fiscal year; NH, Native Hawaiian; OMOP, Observational Medical Outcomes Partnership; OPI, Other Pacific Islander; PSSG, Planning Systems Support Group; UC, urgent care.

© 2025 Ramachandran A et al. *JAMA Network Open*.

eTable 3. Characteristics of Low-Acuity ED Users Before and After Virtual Care Expansion

	**Baseline Year 3**		**Expansion Year 3**		**SMD**
	N, %	%	N	%	
Total	658,461 (100%)	100.0	584,039	100.0	
Age Median [IQR]	60 [46-70]	46-70	61	46-72	0.04
Age Category					0.05
18-45	158,599 (24.1%)	24.1	143,265	24.5	
46-65	250,037 (38.0%)	38.0	207,825	35.6	
66+	249,720 (37.9%)	37.9	232,847	39.9	
Male	579,528 (88.0%)	88.0	508,320	87.0	0.03
Race					0.04
American Indian or Alaska Native	5,447 (0.9%)	0.9	4,932	0.9	
Asian	6,371 (1.0%)	1.0	6,550	1.2	
Black or African American	188,505 (30.4%)	30.4	174,462	32.1	
Native Hawaiian or Other Pacific Islander	5,617 (0.9	0.9	5,331	1.0	
White	414,083	66.8	351,733	64.8	
Ethnicity = Not Hispanic or Latino (%)	595,680	92.4	518,847	91.5	0.04
Service Connection					0.18
None	226,991	34.5	168,303	28.8	
0-49%	134,403	20.4	107,460	18.4	
50-99%	198,922	30.2	186,524	32.0	
100%	96,174	14.6	118,178	20.2	
Missing	1,933	0.3	3,202	0.5	
Priority Category					0.16
Highly disabled	316,983	48.1	321,230	55.0	
Low/moderate disability	131,545	20.0	107,831	18.5	
Low-Income	137,379	20.9	91,021	15.6	
Non-Disabled, copayment required	72,516	11.0	63,585	10.9	
Unhoused	59,262	9.0	52,563	0.9	0.02
Mean Elixhauser Comorbidity Score [SD]	4.2	2.9	3.8	2.5	0.13
Mean Driving Miles from nearest VA ED [SD]	29.1	34.3	27.5	32.4	0.05
US Census Region					0.03

© 2025 Ramachandran A et al. *JAMA Network Open*.

Midwest	130,745	19.9	115,376	19.8	
Northeast	67,156	10.2	55,625	9.5	
South	309,405	47.1	280,596	48.1	
West	149,998	22.8	131,223	22.5	
Mean Area Deprivation Index [SD]	55.4	25.5	54.6	25.5	0.03

SMD=standardized mean difference. Per conventional guidance, SMD>0.1 indicates a meaningful difference between groups.

© 2025 Ramachandran A et al. *JAMA Network Open*.

eTable 4. Sensitivity Analysis of Estimated Change in VA ED Utilization Following a 10% Increase in Alternative Care Use

	**Incidence Rate Ratio (VA ED visits)**	**95% CI**	**p-value (Wald)**
**Virtual Visits (10% increase)**	0.998	0.997 - 0.999	0.003
**Community Visits (10% increase)**	0.989	0.988 - 0.990	<0.001
**In-Person Visits (10% increase)**	1.013	1.012 - 1.015	<0.001

Results from a Generalized Linear Model with a random effect for VA facility. Model includes offset for number of enrollees per facility and interaction for pre vs. post virtual care expansion. Results are scaled to represent the estimated change in VA ED utilization per 10% increase in alternative care, per month and VA facility.

© 2025 Ramachandran A et al. *JAMA Network Open*.

eFigure. Trends in ED Utilization Following Virtual Care Expansion Among 4 Low-Acuity Diagnoses

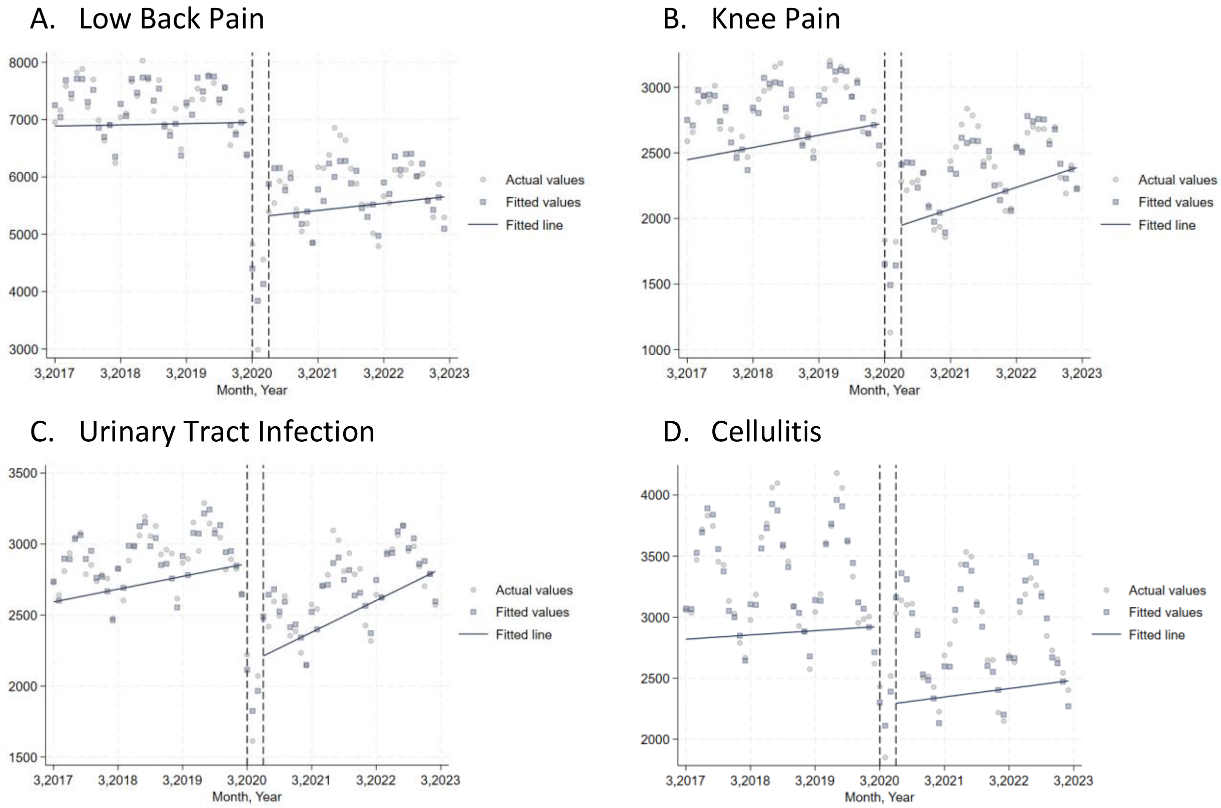

© 2025 Ramachandran A et al. *JAMA Network Open*.
