# Supplementary material for: Emergency Department Utilization by Veterans for Low-Acuity Conditions After Virtual Care Expansion
Source: JAMA Netw Open. 2025 Nov 26;8(11):e2545696. doi: 10.1001/jamanetworkopen.2025.45696 (PMC12658669; doi:10.1001/jamanetworkopen.2025.45696)
Supplement: Supplement 2. — Data Sharing Statement [file jamanetwopen-e2545696-s002.pdf]

Data Sharing Statement

Ramachandran. Emergency Department Utilization by Veterans for Low-Acuity Conditions After Virtual Care Expansion. *JAMA Netw Open*. Published November 26, 2025. doi:10.1001/jamanetworkopen.2025.45696

Data
Data available: No

Additional Information
Explanation for why data not available: US Department of Veterans Affairs (VA) regulations and our ethics agreements require that the analytic data sets used for this study not leave the VA firewall without a Data Use Agreement. This limitation is consistent with other studies based on VA data. However, VA data are made freely available to researchers with an approved VA study protocol. For more information, please visit https://www.virec.research.va.gov
